# Supplementary material for: Pyrosequencing Analysis of O-6-Methylguanine-DNA Methyltransferase Methylation at Different Cut-Offs of Positivity Associated with Treatment Response and Disease-Specific Survival in Isocitrate Dehydrogenase-Wildtype Grade 4 Glioblastoma
Source: Int J Mol Sci. 2024 Jan 3;25(1):612. doi: 10.3390/ijms25010612 (PMC10779484; doi:10.3390/ijms25010612)
Supplement: Supplementary file 1 [file ijms-25-00612-s001.zip › ijms-2786373-supplementary.pdf]

Supplementary Table S1. General Patient Information. M = Male; F = Female; yo = Years-old; WHO = World Health Organization; IDH = Isocitrate Dehydrogenase; MGMT = O-6-Methylguanine-DNA Methyltransferase.

Gender	Age in Diagnosis Moment	MGMT Methylation (%)	MGMT Methylation Range (%)	Diagnosis Day	Death Day	Follow-Up (Days)	DSS	Treatment Response
F	68 yo	21.75	20-23	31\10\2022	Alive	372	Alive	Progressive Disease
F	71 yo	35.0	10-77	31\08\2022	13\11\2022	74	Cancer Death	Death
M	78 yo	3.25	2-5	31\01\2020	18\08\2020	200	Cancer Death	Death
F	65 yo	67.75	45-85	30\12\2019	14\02\2020	46	Cancer Death	Death
F	70 yo	8.75	7-14	30\06\2021	27\07\2021	27	Cancer Death	Death
M	75 yo	5.0	2-8	30\06\2020	06\07\2021	371	Cancer Death	Death
M	63 yo	2.25	1-4	29\06\2021	30\12\2022	549	Cancer Death	Death
M	62 yo	16.0	8-26	29\04\2020	29\10\2020	183	Cancer Death	Death
F	70 yo	4.75	3-7	29\03\2021	13\09\2021	168	Cancer Death	Death
F	57 yo	6.25	5-7	28\11\2022	18\08\2023	263	Cancer Death	Death
F	69 yo	19.0	9-27	28\06\2022	Alive	497	Alive	Good Response to Treatment
F	77 yo	26.25	12-66	28\05\2019	25\10\2019	150	Cancer Death	Death
F	61 yo	40.5	13-52	27\12\2019	09\09\2020	257	Cancer Death	Death
M	70 yo	2.25	1-4	27\08\2021	07\01\2022	133	Cancer Death	Death
F	76 yo	23.25	21-28	25\05\2022	02\08\2022	69	Cancer Death	Death
M	76 yo	65.5	23-85	24\10\2022	22\05\2023	210	Cancer Death	Death
M	66 yo	3.25	2-4	24\05\2022	18\05\2023	359	Cancer Death	Death
M	66 yo	3.25	2-6	24\05\2019	30\06\2019	37	Cancer Death	Death
M	66 yo	4.25	3-5	23\08\2019	12\12\2019	111	Cancer Death	Death
F	60 yo	2.0	2-2	23\06\2022	Alive	502	Alive	Progressive Disease

Gender	Age in Diagnosis Moment	MGMT Methylation (%)	MGMT Methylation Range (%)	Diagnosis Day	Death Day	Follow-Up (Days)	DSS	Treatment Response
F	81 yo	3.25	2-5	23\05\2022	11\08\2022	80	Cancer Death	Death
F	75 yo	33.0	13-81	23\03\2020	16\04\2020	24	Cancer Death	Death
M	75 yo	4.0	3-6	22\05\2020	11\03\2022	658	Cancer Death	Death
M	70 yo	2.25	2-3	22\04\2020	07\02\2021	291	Cancer Death	Death
M	61 yo	10.0	4-15	20\10\2020	01\06\2021	224	Cancer Death	Death
F	76 yo	42.0	9-55	20\08\2019	15\10\2019	56	Cancer Death	Death
F	82 yo	19.25	8-48	20\06\2022	02\03\2023	255	Cancer Death	Death
F	56 yo	75.0	49-100	19\05\2022	25\05\2022	6	Cancer Death	Death
M	57 yo	3.0	2-4	18\12\2020	09\04\2021	112	Cancer Death	Death
M	45 yo	57.5	54-62	18\11\2022	Alive	354	Alive	Good Response to Treatment
M	68 yo	43.5	43-44	18\11\2020	29\12\2021	406	Cancer Death	Death
F	63 yo	16.25	0-61	18\07\2022	05\12\2022	140	Cancer Death	Death
F	65 yo	5.0	4-6	18\05\2020	12\08\2021	451	Cancer Death	Death
F	56 yo	2.25	2-3	18\03\2020	18\05\2020	58	Cancer Death	Death
F	65 yo	5.75	4-9	18\02\2019	03\03\2022	1109	Cancer Death	Death After Recurrence in 03\11\2021
M	75 yo	32.0	11-49	18\01\2019	28\02\2019	41	Cancer Death	Death
M	77 yo	80.5	59-100	17\10\2022	30\06\2023	256	Cancer Death	Death
F	71 yo	10.5	4-22	17\09\2020	02\12\2020	76	Cancer Death	Death
M	46 yo	50.0	46-53	17\03\2022	Alive	600	Alive	Good Response to Treatment
M	67 yo	4.0	2-7	17\02\2021	21\02\2022	369	Cancer Death	Death
F	45 yo	11.5	9-16	16\06\2021	Alive	874	Alive	Good Response to Treatment
M	59 yo	54.25	21-100	14\12\2021	29\06\2022	197	Cancer Death	Death

Gender	Age in Diagnosis Moment	MGMT Methylation (%)	MGMT Methylation Range (%)	Diagnosis Day	Death Day	Follow-Up (Days)	DSS	Treatment Response
M	71 yo	4.25	3-6	14\09\2019	10\08\2020	331	Cancer Death	Death
M	75 yo	20.0	13-28	14\01\2020	31\12\2020	352	Cancer Death	Death
F	65 yo	8.25	4-16	13\05\2019	12\04\2020	335	Cancer Death	Death
M	69 yo	2.5	2-4	13\01\2022	18\04\2023	460	Cancer Death	Death
M	63 yo	13.75	7-23	13\01\2020	25\02\2021	409	Cancer Death	Death
F	59 yo	15.25	13-21	12\12\2019	15\03\2020	94	Cancer Death	Death
M	48 yo	9.75	8-11	12\04\2022	10\10\2023	543	Cancer Death	Death
F	86 yo	43.75	19-76	11\10\2021	23\11\2021	43	Cancer Death	Death
M	74 yo	4.0	2-7	11\09\2019	21\10\2019	40	Cancer Death	Death
F	61 yo	8.0	7-9	11\04\2022	21\06\2022	71	Cancer Death	Death
M	54 yo	2.5	1-4	11\03\2020	02\08\2021	509	Cancer Death	Death
M	39 yo	40.0	14-51	11\02\2021	27\07\2023	896	Cancer Death	Death
M	76 yo	44.75	39-53	11\01\2020	25\05\2020	135	Cancer Death	Death
F	72 yo	2.75	2-4	10\12\2021	08\10\2022	302	Cancer Death	Death
M	68 yo	7.0	6-8	10\10\2019	05\01\2021	453	Cancer Death	Death
M	61 yo	1.5	1-2	10\06\2021	10\05\2022	334	Cancer Death	Death
F	77 yo	29.75	11-51	09\04\2021	16\06\2021	68	Cancer Death	Death
M	61 yo	2.25	1-4	09\03\2020	14\09\2020	189	Cancer Death	Death
M	69 yo	66.25	60-73	08\07\2022	06\02\2023	213	Cancer Death	Death
M	79 yo	32.5	27-39	07\10\2020	02\02\2023	848	Cancer Death	Death
M	65 yo	6.25	5-9	07\07\2020	21\06\2021	349	Cancer Death	Death
F	16 yo	52.75	24-87	07\05\2020	Alive	1279	Alive	Good Response to Treatment

Gender	Age in Diagnosis Moment	MGMT Methylation (%)	MGMT Methylation Range (%)	Diagnosis Day	Death Day	Follow-Up (Days)	DSS	Treatment Response
M	81 yo	2.25	2-3	07\01\2021	20\11\2021	317	Cancer Death	Death
M	53 yo	50.0	49-51	06\11\2020	Alive	1096	Alive	Progressive Disease
M	51 yo	23.75	14-30	06\10\2020	23\01\2022	474	Cancer Death	Death
M	53 yo	19.5	15-27	06\06\2019	23\11\2020	536	Cancer Death	Death After Recurrence in 29\07\2019
M	71 yo	41.75	24-59	06\02\2020	23\06\2021	503	Cancer Death	Death
F	60 yo	3.0	3-3	05\01\2021	11\06\2021	157	Cancer Death	Death
M	30 yo	3.0	2-5	04\10\2021	12\08\2022	312	Cancer Death	Death
F	61 yo	3.2	1-6	04\06\2022	28\09\2022	116	Cancer Death	Death
M	34 yo	7.25	4-12	03\12\2019	29\03\2023	1212	Cancer Death	Death After Recurrence in 08\03\2022
M	58 yo	44.0	39-52	03\05\2019	Alive	1649	Alive	Good Response to Treatment
F	74 yo	5.0	3-9	03\03\2020	26\05\2020	84	Cancer Death	Death
F	77 yo	53.75	13-69	02\07\2021	25\11\2021	146	Cancer Death	Death
M	58 yo	7.25	4-12	01\07\2022	07\10\2023	466	Cancer Death	Death
M	45 yo	3.75	3-5	01\03\2022	Alive	616	Alive	Progressive Disease
